# Supplementary material for: Outpatient Antibiotic and Antiviral Utilization Patterns in Patients Tested for Respiratory Pathogens in the United States: A Real-World Database Study
Source: Antibiotics (Basel). 2022 Aug 4;11(8):1058. doi: 10.3390/antibiotics11081058 (PMC9405217; doi:10.3390/antibiotics11081058)
Supplement: Supplementary file 1 [file antibiotics-11-01058-s001.zip › antibiotics-1831372-supplementary.pdf]

Supplemental Figure S1. Index test positivity rates

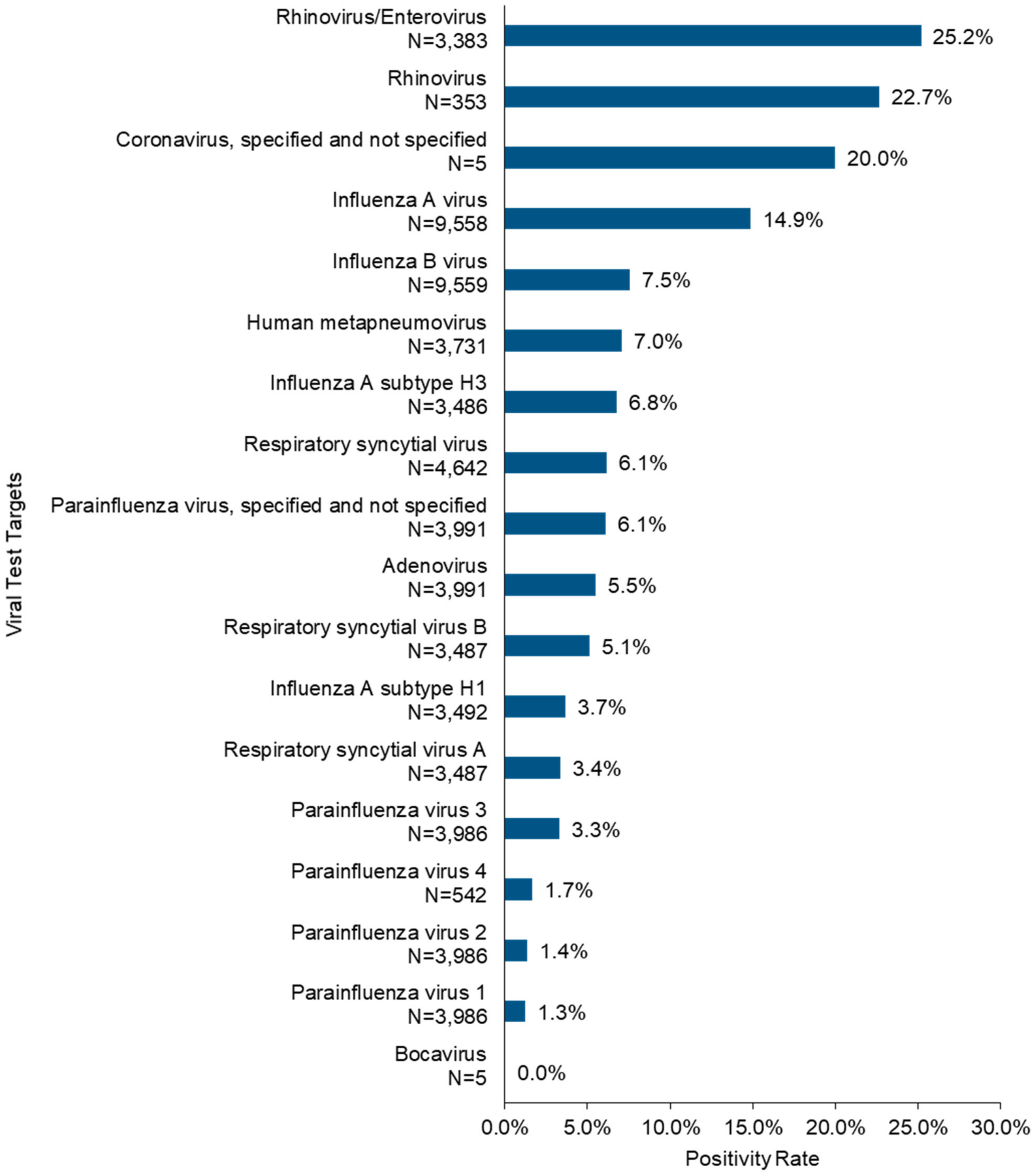

The viral test targets shown in the figure are based on how results were reported for specific tests. For example, in 3,383 tests, rhinovirus/enterovirus was as a single viral target and the result was jointly reported as positive or negative.
